# Direct Recordings of Pitch Responses from Human Auditory Cortex

**DOI:** 10.1016/j.cub.2010.04.044

**Published:** 2010-06-22

**Authors:** Timothy D. Griffiths, Sukhbinder Kumar, William Sedley, Kirill V. Nourski, Hiroto Kawasaki, Hiroyuki Oya, Roy D. Patterson, John F. Brugge, Matthew A. Howard

**Affiliations:** 1 Institute of Neuroscience, Newcastle University Medical School, Framlington Place, Newcastle upon Tyne NE2 4HH, UK; 2 Department of Neurosurgery, University of Iowa Hospitals and Clinics, 200 Hawkins Drive, Iowa City, IA 52242-1061, USA; 3 Centre for the Neural Basis of Hearing, Department of Physiology, Development and Neuroscience, University of Cambridge, Downing Street, Cambridge CB2 3EG, UK

**Keywords:** SYSNEURO

## Abstract

Pitch is a fundamental percept with a complex relationship to the associated sound structure [1]. Pitch perception requires brain representation of both the structure of the stimulus and the pitch that is perceived. We describe direct recordings of local field potentials from human auditory cortex made while subjects perceived the transition between noise and a noise with a regular repetitive structure in the time domain at the millisecond level called regular-interval noise (RIN) [2]. RIN is perceived to have a pitch when the rate is above the lower limit of pitch [3], at approximately 30 Hz. Sustained time-locked responses are observed to be related to the temporal regularity of the stimulus, commonly emphasized as a relevant stimulus feature in models of pitch perception (e.g., [1]). Sustained oscillatory responses are also demonstrated in the high gamma range (80–120 Hz). The regularity responses occur irrespective of whether the response is associated with pitch perception. In contrast, the oscillatory responses only occur for pitch. Both responses occur in primary auditory cortex and adjacent nonprimary areas. The research suggests that two types of pitch-related activity occur in humans in early auditory cortex: time-locked neural correlates of stimulus regularity and an oscillatory response related to the pitch percept.

## Highlights

► We report direct recordings of electrical activity from human auditory cortex ► We distinguish activity related to stimulus regularity and to perceived pitch ► Both are demonstrated in primary cortex and adjacent “core” areas

## Results

**Figure 1:**
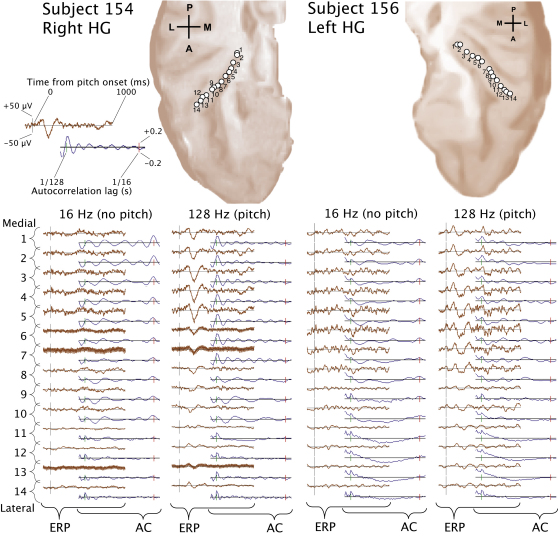
Time-Locked Responses at Different Stimulus Rates The top panels show the superior aspect of the temporal lobes, oriented such that the anterior pole is shown facing downward. Electrode locations (oriented along the axis of Heschl's gyrus [HG]) are superimposed. The lower part of the figure shows the event-related potential (ERP) and the autocorrelation of the ERP (AC) in separate pairs of columns for each subject, where the ERP and AC at each electrode are shown in different colors. Each row corresponds to a different electrode position marked on the sections at the top. The 16 Hz repetition rate does not produce a pitch percept; the 128 Hz repetition rate produces a strong pitch percept. The following abbreviations are used: A, anterior; P, posterior; M, medial; L, lateral.

**Figure 2:**
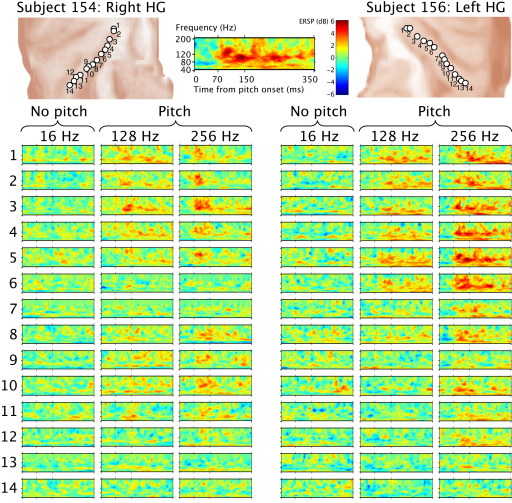
Induced Responses at Different Stimulus Rates Time-frequency analyses showing event-related spectral perturbation (ERSP) corresponding to the transition between noise and the regular-interval noise (RIN) at each electrode in columns that correspond to the 16 Hz rate (in which no pitch is perceived) and the 128 Hz and 256 Hz rates (in which a strong pitch is perceived). A response with a latency of 70 ms is observed in the high gamma range (80–120 Hz), and it is in the same frequency range for pitch values of 128 Hz and 256 Hz. It is not present for the transition between noise and RIN with a rate of 16 Hz (in which pitch is not perceived).

We recorded local field potentials (LFPs) from auditory cortex along Heschl's gyrus (HG) during presentation of a 1 s noise immediately followed by 1.5 s of regular-interval noise (RIN) [2, 4]. RIN is constructed by delaying a copy of a noise waveform for a period of, say, d, and adding it back to the original noise (see Figure S2 available online). When this process is iterated a number of times, the resultant RIN exhibits temporal regularity at the period of the delay, which is heard as a pitch at 1/d, provided that 1/d is greater than the lower limit of pitch, which is about 30 Hz. RIN can be tailored to have the same long-term distribution of energy over frequency and time as the original noise, so it is an excellent stimulus for isolating neural activity associated with the processing of temporal regularity and the perception of pitch (e.g., [5, 6]). Figure 1 shows time-locked responses for stimuli where 1/d is either 16 Hz (well below the lower limit of pitch) or 128 Hz (well above it); both stimuli are highly regular (constructed with 16 iterations), and so the 128 Hz stimulus produces a strong pitch. The 16 Hz RIN is perceived to repeat, but the perception is more like that of a motorboat rather than a tone. Figure 1 shows evoked potentials corresponding to the transition between noise and RIN, as well as the autocorrelation function of the evoked potential after the transition as a measure of the ongoing regularity in the response. There are peaks in the autocorrelation function corresponding to the stimulus regularity at 1/16 s for the 16 Hz stimulus and at 1/128 s for the 128 Hz stimulus. Figure 2 shows time-frequency analyses (Supplemental Experimental Procedures) to demonstrate induced responses to stimuli with fixed regularity and different rates. Figure 2 shows gamma band activity in the 80–120 Hz range that is present for the two rates associated with pitch (128 Hz and 256 Hz) but not for the rate that is not associated with pitch (16 Hz). The gamma activity starts 50–70 ms after the transition from noise to RIN. It continues while the RIN is present and it has the same form irrespective of the pitch value. There was no low-frequency activity below the gamma range, as reported in previous visual studies [7].

**Figure 3:**
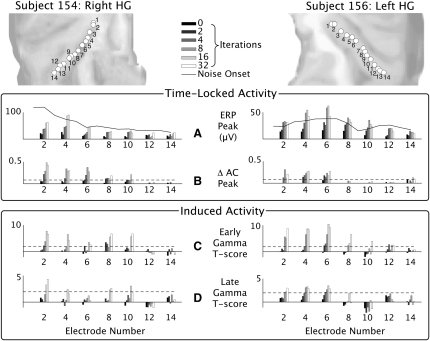
Time-Locked and Induced Activity as a Function of Temporal Regularity Data from the single-rate experiment. (A) Magnitude of the evoked potential at each electrode as a function of stimulus regularity, determined by the number of iterations in the delay-and-add algorithm used to make the stimulus. The onset response to noise is shown as a continuous line for comparison. A fixed pitch value of 128 Hz was used. (B) ΔAC represents the increase in autocorrelation of the response at a lag of 1/128 s, corresponding to the stimulus regularity; the dotted line shows the threshold for a significant autocorrelation response (see Supplemental Experimental Procedures). (C) Increase in induced gamma power between noise and the early RIN response 100–300 ms after transition (expressed as a T score). (D) Increase in induced gamma power between noise and the late RIN response 500 to 1200 ms after transition. The dotted line shows the threshold for significant change in gamma when T = 1.96. The frequency axis shows responses from 40–200 Hz; no induced response was demonstrated below 40 Hz.

**Figure 4:**
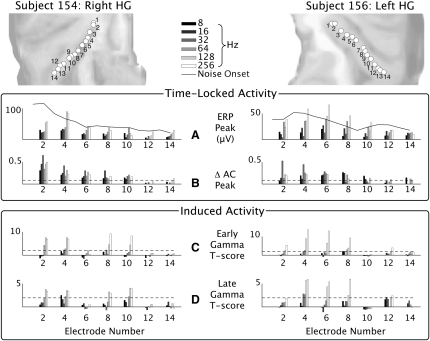
Time-Locked and Induced Activity as a Function of Stimulus Rate Data from the multiple-rate experiment. (A) Magnitude of the evoked potential at each electrode for the transition between noise and RIN with fixed regularity (16 iterations, highly regular) for RIN rates of 8, 16, 32, 64, 128, and 256 Hz. Rates of 8 and 16 Hz are well below the lower limit of pitch, whereas rates of 128 and 256 Hz are well above the limit. (B) Increase in the AC of the local field potential (LFP) at a lag corresponding to the rate of the RIN as a function of rate. (C) Increase in gamma power between noise and the early RIN (expressed as a T score). (D) Increase in gamma power between noise and the late RIN as functions of RIN rate.

The systematic effects of stimulus regularity and rate on the time-locked and induced responses are summarized in Figure 3 and Figure 4. The autocorrelation responses summarized in Figure 3B and Figure 4B are measures of ongoing regularity in the stationary evoked potential between 500 ms and 1200 ms after transition. The autocorrelation value at the lags corresponding to the stimulus rates is summarized. The early-induced gamma activity (100 to 300 ms) is summarized in Figure 3C and Figure 4C, and the late gamma activity (500 to 1200 ms) is summarized in Figure 3D and Figure 4D (see Supplemental Experimental Procedures and Figure S3 for measurement of induced gamma power changes).

### Relationship of Time-Locked and Induced Responses to the Stimulus Property of Regularity

Figure 3A shows evoked potential magnitudes for the transition between noise and RIN for a fixed pitch value of 128 Hz. The continuous line shows the magnitude of the noise-onset response for comparison. Figure 3B shows the increase in the autocorrelation value (ΔAC) of the LFP at a lag of 1/128 s, because of the transition between noise and RIN, as a function of stimulus regularity (as determined by the number of cycles of the delay-and-add algorithm that was used to construct the stimulus [2]). The dotted line corresponds to the threshold for a significant change in AC (Supplemental Experimental Procedures). Figures 3C and 3D show the increase in induced gamma power between noise and the early and late period of RIN presentation.

Figure 3 shows significant increases in the regularity of the LFPs, as measured by AC, as a function of stimulus regularity in HG. The responses are not transition responses like the evoked potentials and are present 500 ms after transition. Subject 154 shows AC responses that increase as a function of regularity in the electrodes located in medial and middle HG up to electrode 10. These responses are never significant when there is no regularity in the signal and are always significant when there are more than two iterations present (with a single exception at electrode 10 for 32 iterations). The data for subject 156 show a monotonic increase in the AC response in electrodes 4 to 6. These AC responses are ensemble neural responses to stimulus regularity.

Figure 3 also shows an increase in induced activity measured in the high gamma band in the range 80–120 Hz. Significant increases in both early (Figure 3C) and late (Figure 4D)-induced gamma power occur in similar electrodes to the AC responses. Based on these data alone, for the 128 Hz stimulus, it not possible to determine whether gamma is a neural correlate of the stimulus regularity or the perceived pitch.

### Relationship of Time-Locked and Induced Responses to Pitch Value

Figure 4A shows the evoked potential magnitude for the transition between noise and RIN with fixed regularity (16 iterations, highly regular) and repetition rates of 8, 16, 32, 64, 128, and 256 Hz. The first two values (8 and 16 Hz) are well below the lower limit of pitch [3], and the last two (128 and 256 Hz) are well above the limit. Figure 4B shows the increase in the AC of the LFP at lags corresponding to the rate of the RIN as a function of rate. Figures 4C and 4D show the increase in gamma power between noise and the early and late RIN as a function of RIN rate.

Figure 4B shows significant increases in the regularity of the LFP for RIN in HG, irrespective of whether the stimulus rate is below or above the lower limit of pitch. Subject 154 and subject 156 both show significant increases in AC through the range 8–256 Hz up to electrode 10, with more variability in the responses of subject 156. This provides further support for the representation of stimulus regularity in neural ensembles in early auditory cortex that can be measured by AC.

Figures 4C and 4D show significant increases in induced gamma activity in the LFP in the 80–120 Hz range to occur both early (100 to 300 ms) and late (500 to 1200 ms) in HG when the regularity of the stimulus is at, or above, the lower limit of pitch. All of the significant early and late gamma responses occur at 32 Hz or above, with the exception of the late gamma response in electrode 4 for the 8 Hz rate in subject 154. The detailed form of the relationship between stimulus rate and gamma power is given in Figure S1.

### Anatomical Substrate for Responses

In both subjects, the significant regularity and induced gamma responses occur only between electrodes 1 and 10; see Tables S1 and S2 for Talairach coordinates and Supplemental Experimental Procedures for a detailed anatomical discussion. Electrode 1 is in the primary cortex and electrode 10 is in a region of central HG, where we see a second maximum in sound-minus-silence contrasts in fMRI work [5].

## Discussion

In this human study, stimulus representations are distinguished from perceptual representations in early auditory cortex. Direct recording of LFPs reveals a representation of stimulus regularity in the evoked responses and induced gamma responses related to the perception of pitch. The magnitude of the regularity response increases as the regularity of the RIN increases, and it continues throughout the duration of the RIN. Significant regularity responses occur both above and below the lower limit of pitch. Induced oscillatory responses are demonstrated in similar areas. These oscillations are significantly different from those occurring during the presentation of the preceding noise when the stimulus rate is above the lower limit of pitch. The responses to regularity demonstrated by autocorrelation are not transition responses but are steady-state responses that are present in the LFP more than 500 ms after pitch onset. Likewise, the gamma responses persist for the duration of the stimulus. Both responses occur in primary and adjacent nonprimary cortex.

### Relationship to Animal Recordings from Single Neurons

These human LFPs are ensemble responses from dendrites, as opposed to previous single-neuron recordings of spike activity [8, 9]. Whether pitch representation requires single neurons or ensembles is debatable: consider spatial perception, for which single neurons are not adequate [10, 11]. Nor is it clear whether the same pitch mechanism should exist in more than 200 primate species, let alone more distant species.

Previous marmoset recordings [8] from a subarea abutting primary cortex showed responses in a proportion of neurons that were better defined in the pitch than the frequency domain. The study used a missing-fundamental stimulus, and the responses can be regarded as tuned in the domains of either stimulus regularity or perceived pitch. Using a less strict criterion for pitch tuning, a ferret study [9] showed an effect of pitch value on responses in multiple cortical areas. That study used spectrally shaped harmonic sounds, and it also cannot be uniquely interpreted in terms of stimulus or pitch representation.

The present study shows that responses to both stimulus regularity and perceived pitch occur in neural ensembles in primary cortex and in an adjacent area. In the event that a universal mechanism for pitch analysis was to exist, these data would not localize it to one subregion. These rare human recordings do not allow sampling from the same number of auditory areas as does the ferret work.

### Relationship to Human Studies

One previous report describes direct pitch recordings from a part of HG [12] via a lateral approach. A previous study of ensemble activity, measured with magnetoencephalography, showed activity in medial HG corresponding to the transition between noise and RIN associated with pitch [6]. The responses in that study are transition responses to the onset of pitch similar to the event-related potentials (ERPs) here. The current ERP study allows the direct localization of such ERPs to HG without source modeling. The maximal ERP responses in this study occur in the medial half of HG, consistent with the previous study.

Ensemble activity to pitch-producing stimuli, measured indirectly with the fMRI BOLD response, demonstrates maxima in a part of HG that is more lateral than the gamma activity reported here [5, 13]. However, it should be noted that there is overlap of the more medial activity in the Patterson et al. study with the gamma activity reported here (Supplemental Experimental Procedures). The present finding that the maximal sensory and perceptual pitch responses are found medial to the BOLD maxima in the previous studies requires explanation, given the coupling between LFPs and BOLD shown in other studies [14, 15]. One possible factor is the anatomical variability in lateral HG that might affect the electrode sampling, an issue that could be addressed by carrying out fMRI and depth electrode recording in the same subjects.

Hall and Plack [16] recently suggested that identification of pitch processing centers in the brain requires pitch-related activity in the same region of the cortex for a wide range of pitch-producing stimuli (see Supplemental Discussion). To satisfy such a conservative criterion, the current findings would require extension using different pitch-evoking stimuli. The current data, which suggest yoking between the anatomical position of a stimulus correlate and the associated perceptual correlate, raise the intriguing possibility that neural correlates of the pitch percept in early auditory areas might not be invariant with respect to the stimulus with which they are associated. If that were to be the case, it would call into question any obligatory requirement for neural correlates of pitch to be invariant with respect to the form of the stimulus.

### Conversion of Stimulus Mapping to Pitch Mapping

The data show that responses related to regularity and to perceived pitch are mapped to similar areas. This might be achieved within those areas by conversion of relevant stimulus parameters into a neural correlate of the percept. This is not the only mechanism, however, and another possibility is that higher areas that interpret the stimulus might cause local activity that is associated with the percept. Such a mechanism would be consistent with constructive models of auditory perception [17] that require top-down connections from nonprimary cortex. Such mechanisms have been previously suggested in both the visual [18] and auditory systems [19].

## Supplemental Information

Document S1. Supplemental Data, Supplemental Experimental Procedures, Three Figures, and Two Tables
