# Supplementary material for: Direct Recordings of Pitch Responses from Human Auditory Cortex
Source: Curr Biol. 2010 Jun 22;20(12):1128–32. doi: 10.1016/j.cub.2010.04.044 (PMC3221038; doi:10.1016/j.cub.2010.04.044)
Supplement: Document S1. Supplemental Data, Supplemental Experimental Procedures, Three Figures, and Two Tables [file mmc1.pdf]

Current Biology, Volume *20*

Supplemental Information

Direct Recordings of Pitch Responses from Human Auditory Cortex

T. D. Griffiths, S. Kumar, W. Sedley, K. Nourski, H. Kawasaki, H. Oya, R. D. Patterson, J. F. Brugge, and M. A. Howard

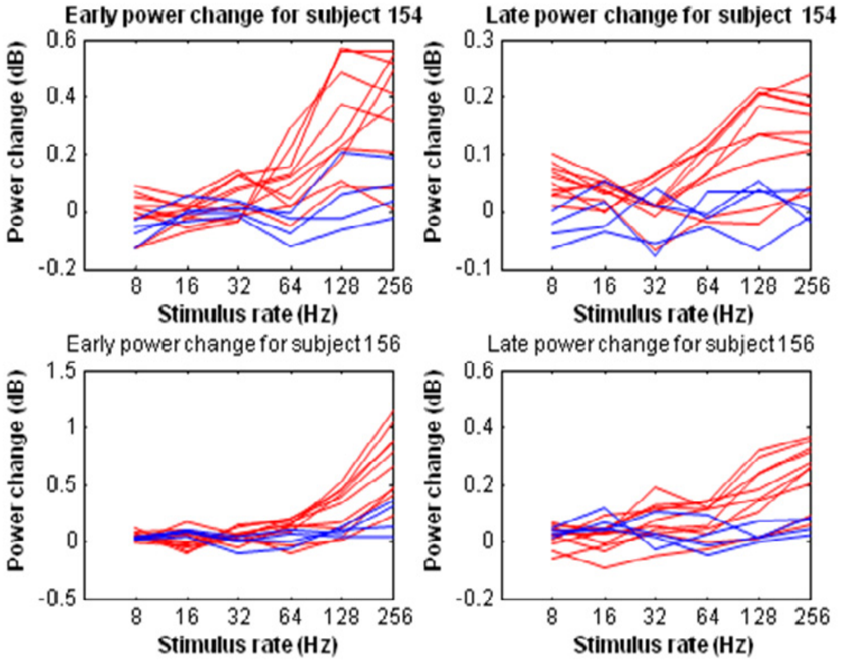

Figure S1. Form of Relationship between Stimulus Rate and Gamma Power
Early (100ms to 300ms after transition) and late (500ms to 1200ms after transition) power changes in subjects 154 and 156 as a function of pitch value. The plots correspond to the same power values that are used to calculate significant changes in power in Figures 4c and 4d. Note that plots are shown for all 14 electrodes here (Figures 4c and 4d show alternate electrodes for clarity).The red plots correspond to the medial electrodes (1 - 10) where significant increases in gamma power are demonstrated in Figures 4c and 4d. The blue plots correspond to the lateral electrodes (11 – 14).

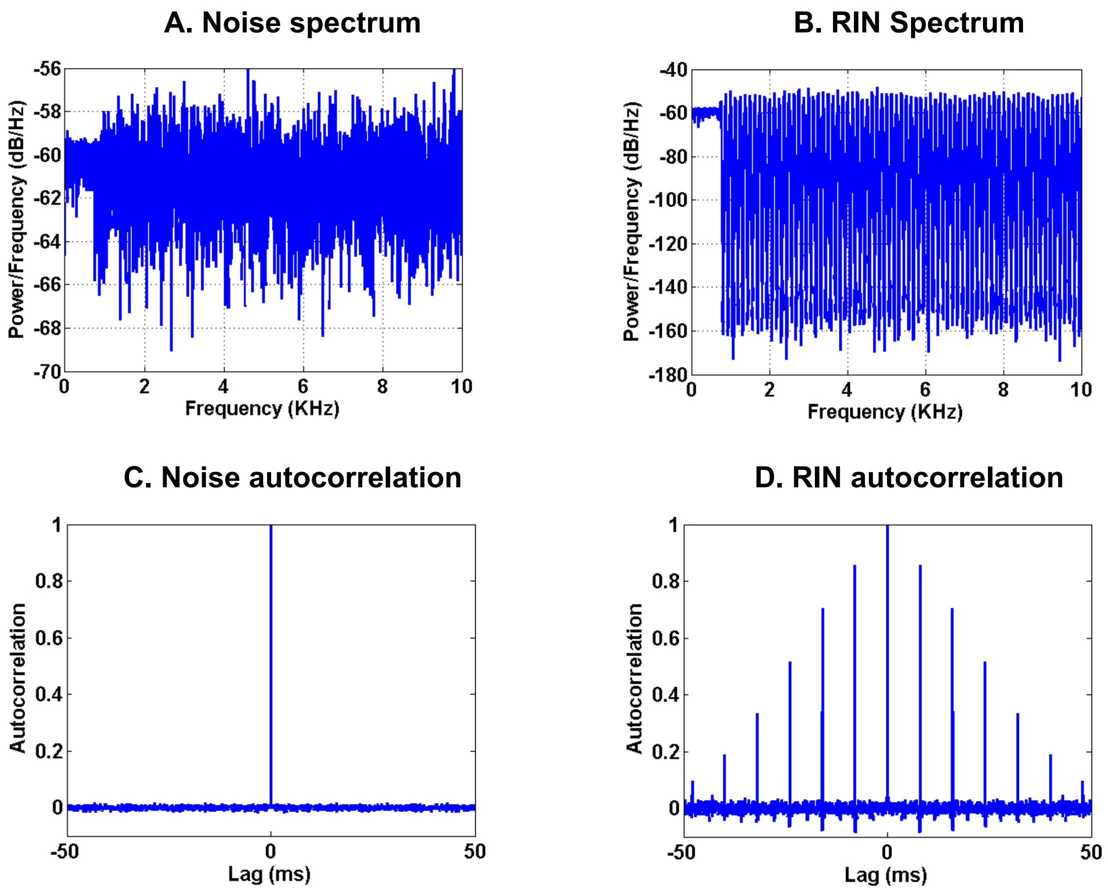

Figure S2. Acoustic Properties of Stimulus

Spectral and temporal properties of the stimulus. (A) Power spectral density of the noise segment before RIN onset, (B) Power spectral density of the RIN, (C) Autocorrelation function of noise segment before RIN onset, and (D) Autocorrelation function of the RIN. The RIN in B and D has a rate of 128 Hz and is made from a random noise using a delay-and-add algorithm with 16 iterations. This imparts a high degree of regularity as illustrated by the large peak in the autocorrelation function (D) at a lag of 1/128 s (a RIN delay of approximately 8 ms). When the regularity is high, secondary peaks are also seen at integer multiples of this lag.

Psychophysical tests show that the pitch strength of the RIN correlates with the log of the magnitude of the first peak of the autocorrelation function [6].The regularity of the RIN is present irrespective of whether the pitch value is above the lower limit of pitch or below it. In the experiment, we employ the same autocorrelation process to demonstrate that there is strong temporal regularity in the time-locked neural responses recorded from auditory cortex, independent of whether the rate is above or below the lower limit of pitch. In contrast, we also show that induced oscillatory responses are only significant for stimuli with rates above the lower limit of pitch, suggesting that the oscillatory response is a better candidate for a neural correlate of pitch perception.

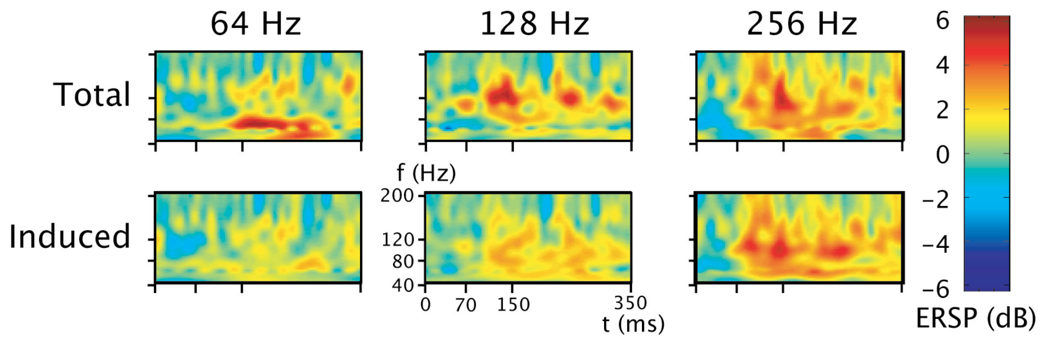

Figure S3. Effect of ERP Subtraction on Induced Responses
The figure shows the effect of ERP subtraction on the induced responses at the three rates shown in Figure 2 in the main manuscript. The data are from electrode 4 of subject 156. The top row shows the total responses based on time-frequency analysis and the bottom row the induced responses: the removal of the time-locked response to regularity can be seen at 64 Hz and 128 Hz. In this report we use the time-frequency analysis after subtraction of the ERP to define an induced response with no overlap with the time-locked response to stimulus regularity.

Table S1. Talaraich Coordinates [18] of High Impedence Electrodes in Right Auditory Cortex for Subject 154

Electrode	x (mm)	y (mm)	z (mm)
1	30.6	-26.7	15.0
2	32.0	-26.4	14.6
3	33.80	-23.8	12.2
4	35.4	-22.5	10.9
5	36.9	-21.0	9.5
6	38.5	-19.8	8.4
7	41.5	-17.4	6.8
8	43.0	-16.3	6.4
9	44.6	-15.3	6.2
10	46.0	-14.2	5.9
11	48.7	-11.8	4.7
12	49.9	-10.5	3.9
13	51.1	-9.1	3.0
14	52.2	-7.7	2.2

Table S2. Talaraich Coordinates [18] of High Impedence Electrodes in Left Auditory Cortex for Subject 156

Electrode	x (mm)	y (mm)	z (mm)
1	-32.3	-29.0	12.1
2	-32.8	-28.6	11.9
3	-36.7	-26.0	10.6
4	-38.8	-24.6	9.9
5	-40.9	-23.2	9.26
6	-42.9	-21.7	8.6
7	-45.9	-18.5	7.5
8	-47.0	-16.5	7.2
9	-47.9	-14.6	6.8
10	-48.8	-13.0	6.4
11	-50.8	-11.5	4.5
12	-51.9	-11.7	3.1
13	-52.9	-12.1	1.6
14	-54.0	-12.6	0.0

Supplemental Introduction

Study Basis: Distinguishing Sensory and Perceptual Mapping

Pitch is a fundamental percept with a complex relationship to the stimulus: early theory [1] emphasised stimulus frequency composition, whilst current models emphasise stimulus regularity. Thus, a simple relationship between cortical areas (defined by mapping of the stimulus property of frequency) and any neural correlate of the pitch percept would not be predicted; indeed, the neural basis of the pitch percept is currently highly controversial. In the marmoset, single neurons in a low-frequency area abutting primary auditory cortex, A1, can be more highly tuned to the pitch value of a stimulus rather than the frequency composition of that stimulus [2]. In contrast, ferret studies [3] demonstrate single neurons distributed throughout several cortical areas in which firing is influenced by pitch value. In humans, time-locked activity in neural ensembles related to pitch onset is shown by MEG [4] in medial Heschl's Gyrus (HG), in the region of primary cortex. In contrast, studies based on the BOLD hemodynamic response in response to stimuli associated with pitch [5] show activity in a more lateral area of HG, in non-primary cortex.

Part of the current controversy concerning the neural basis of pitch perception might reflect an ill-posed question: pitch-related activity in the brain might reflect processing of stimulus regularity at a stage before the neural activity associated with the pitch percept itself. No study of brain responses to sounds associated with pitch can ever be uniquely interpreted in terms of sensory representation of stimulus properties relevant to pitch, as opposed to neural correlates of pitch. However, the analysis of responses to stimulus properties across pitch boundaries allows us to search for response changes that might be explained by perceptual rather than stimulus change. In the current study, we assess responses to stimuli with repetition rates that cross the lower boundary for the perception of pitch in an effort to find a neural correlate of pitch in the form of a response that appears in the region of the pitch boundary. This stands in contrast to activity associated with the processing of relevant stimulus features such as regularity; the neural correlate of the percept should only be present when pitch is perceived.

The study employs stimuli composed of a noise followed seamlessly by a temporally regular sound that produces a pitch. At the level of the stimulus, there is a transition from a temporally irregular to a temporally regular stimulus, where the degree of regularity can be quantified by the height of the first peak in the autocorrelation function of the stimulus wave [6]: see Figure S2. When the interval rate for the regular-interval noise is above the lower limit of pitch, the transition from an irregular to a regular stimulus produces a perceptual transition from noise to pitch. Previous psychophysical work [6] has shown a strong correlation between the value and strength of the pitch that is perceived and the rate and degree of temporal regularity of the stimulus. The pitch value (quantified in terms of the repetition rate of a matching click train) equals the repetition rate of the stimulus. The pitch strength has been shown to correlate linearly with the stimulus regularity as measured by the log of the magnitude of the first peak in the autocorrelation function.

The response in this case is a direct recording of the local field potential (LFPs) in primary auditory cortex and adjacent non-primary areas. Time-locked activity in such responses might relate to relevant stimulus attributes such as regularity, which are revealed in the current analysis by calculating the autocorrelation of the LFP wave. Time-frequency analyses were also be carried out on the LFPs to reveal oscillatory activity of a form that has been argued to be a neural correlate of perception in other sensory systems eg [7-11]. Both types of response are

reported for RIN sounds below and above the lower limit of pitch to distinguish neural responses that only arise when humans hear a pitch percept.

Supplemental Results

Further Analysis of Relationship between Stimulus Rate and Gamma Power

Figure 4c and 4d in the manuscript show the significance of change in gamma power between noise and RIN at different stimulus rates at the different electrodes. The actual gamma power change is plotted below in Figure S1 for each electrode as a function of stimulus rate for the interested reader. As expected based on the significant changes in gamma power above the lower limit of pitch (approximately 30Hz) in Figure 4c and 4d, power increases are shown above 32Hz in both subjects. The largest power increases shown in Figure S1 occur in the medial electrodes 1 - 10 where significant responses are demonstrated in Figures 4c and d. The exact form of the relationship between stimulus rate and gamma power above the lower limit of pitch is not consistent between these subjects and requires further clarification in future studies.

Supplemental Discussion

Future Directions and Predictions

a) Use of Other Pitch-Associated Stimuli

A recent human experiment based on the fMRI BOLD response uses an alternative approach to the search for activation associated with pitch [12]. The approach seeks mechanisms related to the pitch produced by a range of different stimuli – activity that would indicate the presence of a single 'pitch centre'. Hall and Plack [12] make a good point that adequate characterisation of a pitch mechanism must explain the pitch associated with a wide range of stimuli. There are issues related to this approach, however. Firstly, the current data suggest gamma activity as a correlate of the percept that is 'yoked' to relevant stimulus attributes in early cortex where the gamma might not be the same for any given pitch-producing stimulus. Secondly, the group level analysis in the previous study requires exact alignment of the pitch mechanism with respect to macroscopic anatomical boundaries for all of the subjects, whilst individual responses are quite variable. Finally, the approach in [12] is based on a range of stimuli including a dichotic Huggins pitch which is not universally perceived (subjects had to be excluded who did not hear it). Moreover, when it is perceived, it is commonly lateralized for reasons that are not at all clear. Whilst the Hall and Plack work suggests common pitch activity posterior to HG in the planum temporale, subsequent work using a refined Huggins pitch that is not lateralised in space [13] demonstrates activity in lateral HG.

The current work suggests that gamma activity is a neural correlate of early pitch analysis that can be measured in individual subjects and tested with other stimuli using other types of perceptual boundary in future experiments.

b) Precise Mapping of the Lower Limit of Pitch

In this work we have systematically mapped responses to different regularity rates between 8 Hz and 256 Hz in octave intervals. The stimuli with a rate of 32-Hz and above are all above the lower limit of pitch, which is approximately 30 Hz [14] [15]. All of the significant gamma

responses reported in this study, with a single exception, occurred within the existence region for pitch.

The previous study of Presnitzer et al. [15] used an objective task to establish the lower limit of pitch based on whether the perceived pitch supports detection of a change in a short melody – emphasised as a critical aspect of the presence of pitch in the American National Standards Institute 1960 definition. A similar limit was defined by Krumbholz et al [14] using an alternative method based on pitch rate discrimination. RIN pitch values of 32 Hz and above are above the 30-Hz limit and they supported the perception of melody for both subjects.

The present data therefore suggest that the gamma response is a good candidate for a neural correlate of pitch. Further testing of this hypothesis will require exact measurement of the lower limit of pitch in individual subjects and the measurement of gamma response across the precise perceptual boundary.

c) Pitch and Timbre Responses

The transition between noise and RIN also produces a perceptual transition below the lower limit of pitch: subjects perceive a periodic fluctuation in the RIN which would normally be classified as a change in the timbre of the sound rather than its pitch. Such stimuli are not associated with pitch perceptions and changes in the RIN rate cannot be used to construct melodies. Modelling studies of RIN [12] have demonstrated acoustic fluctuations in RIN stimuli at the level of hundreds of milliseconds, and Hall and Plack have argued that this attribute, relevant to timbre rather than pitch, explains aspects of the measured neural responses to RIN. However, such fluctuation occurs both below and above the lower limit of pitch. The current study shows gamma responses to RIN that occur reliably with a latency of 70ms, before such fluctuation might be established, and only above the lower limit of pitch. Systematic studies of the neural bases for timbre perception (see [16] for review) demonstrate critical mechanisms in the superior temporal plane beyond HG.

It will be interesting to see whether gamma responses to timbre are discovered, and whether they are observed in primary cortical areas, like the pitch responses reported here.

d) Recording from Other Areas

The current recordings were carried out in two subjects with optimal electrode penetration to allow high impedence recording from human auditory cortex along HG. Some of our neurosurgical subjects undergo recordings from planum temporale and we are interested to record gamma responses from this area in future. The current data, however, suggest that these gamma response are confined to primary cortex and immediately adjacent cortex in HG, and do not extend even to lateral HG.

Supplemental Experimental Procedures

Subjects, Surgery and Recording Electrodes

The two subjects were adults undergoing depth-electrode recording to localise epileptic foci before consideration of surgery. Research protocols were approved by University of Iowa Human Subjects Review Board. Prior informed consent was obtained from each subject before the study. Both subjects had normal hearing as confirmed by audiometric testing prior to implantation of electrodes. Hybrid depth electrodes [17] with 14 high impedance contacts (70 - 300 kΩ) were implanted along the long axis of HG on one side. The electrode contact positions were determined by co-registering electrode locations identified on postoperative MRI scans with each subject's pre-operative 3-dimensional brain MRI. Tables S1 and S2 give the position of the electrodes referenced to Talairach space [18] to allow comparison of the electrode positions with data from imaging studies. The positions were calculated by a further coregistration of the MRI containing the electrode positions with a standard structural template.

Standardised Location of Recording Electrodes

In both subjects, the significant regularity and induced gamma responses occur only between electrodes 1 and 10. In subject 154, the Talairach coordinates [18] were [30.6 -26.7 15.0] and [46.0 -14.2 5.9] for electrodes 1 and 10. In subject 156, the coordinates were [-32.2 -29.0 12.0] and [-48.8 -13.0 6.4]. Electrode 10 is in the region of central HG (see [5]: Table 1) and is in the region where we see a second maximum (lateral to the most medial maximum) in sound-minus-silence contrasts in fMRI work. The data therefore suggest significant regularity and pitch mapping in primary auditory cortex, in medial HG and in an adjacent area in HG. The adjacent area might correspond to primate area R [19].

The peak RIN-versus-noise BOLD contrasts in the previous fMRI study of Patterson et al [5] occurred in lateral HG ([57.2 -8.8 -1.9] and [-55.3 -8.8 1.5]) at a point that would be lateral to electrode 14 in both of the current subjects. Note, however, that the previous contrast was also significant in more medial positions overlapping the electrodes demonstrating significant gamma responses (see previous Figure 2E from [5]).

Stimulus

Subjects listened to stimuli comprising a 1-s burst of broadband noise preceding 1.5 s of RIN. RIN was created using a delay-and-add algorithm [6]. The RIN is also known as iterated rippled noise because of the ripples that are present in the acoustic spectrum. We use the term RIN here to emphasise the temporal cue in the stimulus. The delay in the delay-and-add cycle determines the pitch value, and the number of cycles (iterations) determines the pitch strength or salience. The stimuli were normalised to a common power spectral density, and high-pass filtered at 800 Hz to remove spectral ripples that might be resolved in the cochlea. Broadband noise was also added below the cut-off frequency to ensure that the responses were not induced by cochlear distortion products.

Paradigm

Recordings were made in a dedicated recording facility incorporating a shielded room. Experiments were based on a passive listening paradigm. Subjects were awake and relaxed during the recording sessions. The stimuli were delivered diotically via Etymotic ER4B

earphones in custom earmolds at a comfortable level of 45-55 dB above threshold. In a *single-pitch* experiment, subjects listened to RIN with a repetition rate of 128 Hz constructed with 0, 2, 4, 8, 16, or 32 iterations (50 trials/condition: random order). In a *multiple-pitch* experiment, subjects listened to RIN with 16 iterations and a repetition rate of 8, 16, 32, 64, 128, or 256 Hz (50 trials/condition: random order). Data were acquired from high impedance electrodes at a sample rate of 24414 Hz (down sampled to 1 kHz for analysis) with a resolution of 16 bits. The analysis was carried out after removal of the DC component and any line noise.

Analysis

The data were divided into trials that included a 0.3-s prestimulus interval and a 2.8-s poststimulus interval. Rejection criteria were based on means and standard deviations (SD) across trials. Any trial in which the amplitude exceeded more than 5 SD was rejected. In addition every trial was visually inspected to check for any artefact or electrical interference.

Time-locked activity was analysed in two ways. 1) Average evoked potentials were determined for both noise and RIN onset by taking the average across trials of the time waveform at each electrode. Summary values presented in this report are based on the magnitude of the first negative peak (greatest negative value within 350ms), though the first and second positive and negative peaks all showed concordant trends. 2) Autocorrelation functions of the average evoked potential were calculated. Since the spontaneous activity in intracranial recording is characterized by a 1/f distribution, most spontaneous power is concentrated at lower frequencies, which makes detection of low frequency signals in this background difficult. Accordingly, the raw, average evoked potential was 1/f whitened [20] by taking the first-order differential of the average evoked potential. These potentials were also notch filtered at the odd harmonics of 60 Hz to remove line noise. The autocorrelation function of the evoked potential was computed after the line noise removal and 1/f whitening. A significant change in autocorrelation was defined as 2*(no_of_samples)^-0.5: in a random time series the auto-correlation coefficient (at any lag) is normally distributed with a mean of 0 and variance of 1/N where N is the number of samples in the time series [21].

Non-time-locked activity was assessed using time-frequency analysis which identified pitch related activity in a gamma band from 80- 120Hz (see Figure 2). The average evoked potential was first subtracted from each trial [9, 22]. This was done so that the time frequency analysis identifies induced activity without the time-locked activity related to the stimulus that we seek to distinguish in this study. Figure S3 shows the effect of evoked potential subtraction. The remaining signal in each trial was convolved with a Morlet wavelet [7]. The frequency range was restricted to 14-300 Hz. The number of cycles for the Morlet wavelet varied from 3 (at 14 Hz) to 32 (at 300 Hz). The results are plotted in Figure 2 as event-related spectral perturbations, defined as the median power across trials in dB, at each time-frequency point relative to the baseline power at the relevant frequency toward the end of the noise response. Power change is quantified as a T value associated with the difference power between the 80 – 120 Hz gamma band and the power in the same band toward the end of the noise response (last 200ms of noise presentation before transition). The measure is applied to both the early (100 – 300 ms) and the late (500 – 1200 ms) pitch response.

Supplemental References

1. von Helmholtz, H.L.F. (1885). On the Sensations of Tone, 4th (English translation 1912) Edition, (London: Longmans).
2. Bendor, D., and Wang, X. (2005). The neuronal representation of pitch in primate auditory cortex. Nature *436*, 1161-1165.
3. Bizley, J.K., Walker, K.M.M., Silverman, B.W., King, A.J., and Schnupp, J.W.H. (2009). Interdependent Encoding of Pitch, Timbre, and Spatial Location in Auditory Cortex. J. Neurosci. *29*, 2064-2075.
4. Krumbholz, K., Patterson, R.D., Seither-Preisler, A., Lammertmann, C., and Lutkenhoner, B. (2003). Neuromagnetic evidence for a pitch processing center in Heschl's gyrus. Cereb Cortex *13*, 765-772.
5. Patterson, R.D., Uppenkamp, S., Johnsrude, I.S., and Griffiths, T.D. (2002). The processing of temporal pitch and melody information in auditory cortex. Neuron *36*, 767-776.
6. Yost, W.A., Patterson, R., and Sheft, S. (1996). A time domain description for the pitch strength of iterated rippled noise. J Acoust Soc Am *99*, 1066-1078.
7. Tallon-Baudry, C., and Bertrand, O. (1999). Oscillatory gamma activity in humans and its role in object representation. Trends Cogn Sci *3*, 151-162.
8. Keil, A., Muller, M.M., Ray, W.J., Gruber, T., and Elbert, T. (1999). Human gamma band activity and perception of a gestalt. J Neurosci *19*, 7152-7161.
9. Crone, N.E., Boatman, D., Gordon, B., and Hao, L. (2001). Induced electrocorticographic gamma activity during auditory perception. Brazier Award-winning article, 2001. Clin Neurophysiol *112*, 565-582.
10. Gross, J., Schnitzler, A., Timmermann, L., and Ploner, M. (2007). Gamma oscillations in human primary somatosensory cortex reflect pain perception. PLoS Biol *5*, e133.
11. Wang, Z., Logothetis, N.K., and Liang, H. (2008). Decoding a bistable percept with integrated time-frequency representation of single-trial local field potential. J Neural Eng *5*, 433-442.
12. Hall, D.A., and Plack, C.J. (2009). Pitch Processing Sites in the Human Auditory Brain. Cereb. Cortex *19*, 576-585.
13. Puschmann, S., Uppenkamp, S., Kollmeier, B., and Thiel, C.M. (2009). Dichotic pitch activates pitch processing centre in Heschl's gyrus. Neuroimage.
14. Krumbholz, K., Patterson, R.D., and Pressnitzer, D. (2000). The lower limit of pitch as determined by rate discrimination. J Acoust Soc Am *108*, 1170-1180.
15. Pressnitzer, D., Patterson, R.D., and Krumbholz, K. (2001). The lower limit of melodic pitch. J Acoust Soc Am *109*, 2074-2084.
16. Griffiths, T.D., Kumar, S., Von Kriegstein, K., Overath, T., Stephan, K.E., and Friston, K.J. (2009). Auditory object analysis. In The Cognitive Neurosciences, M.S. Gazzaniga, ed. (Cambridge, MA: MIT Press), pp. 367-381.
17. Howard, M.A., 3rd, Volkov, I.O., Granner, M.A., Damasio, H.M., Ollendieck, M.C., and Bakken, H.E. (1996). A hybrid clinical-research depth electrode for acute and chronic in vivo microelectrode recording of human brain neurons. Technical note. J Neurosurg *84*, 129-132.
18. Talairach, P., and Tournoux, J. (1988). A stereotactic coplanar atlas of the human brain, (Stuttgart: Thieme).
19. Hackett, T.A. (2007). Organization and correspondence of the auditory cortex of humans and nonhuman primates. In Evolution of the Nervous System, J.H. Kaas, ed. (Oxford: Elsevier), pp. 109-119.
20. Buzsaki, G. (2006). Rhythms of the Brain, (Oxford, UK: Oxford University Press).
21. Chatfield, C. (2001). Time Series Forecasting, (London: Chapman and Hall).

22. Steinschneider, M., Fishman, Y.I., and Arezzo, J.C. (2008). Spectrotemporal analysis of evoked and induced electroencephalographic responses in primary auditory cortex (A1) of the awake monkey. Cereb Cortex *18*, 610-625.
